# Prevalence of depression among Iraqi dentistry students during COVID-19 returning to onsite learning: A cross-sectional study

**DOI:** 10.1192/j.eurpsy.2023.369

**Published:** 2023-07-19

**Authors:** G. Alhashem, A. A. R. Abbas, S. A. J. Ali, A. Almhanna

**Affiliations:** ^1^Pharmacy, AlSafwa University College; ^2^College of Dentistry/ University of Kerbala, Kerbala, Iraq

## Abstract

**Introduction:**

Throughout the COVID-19 pandemic, students were vulnerable to mental health issues and dentistry students were no exception. All Iraqi universities were transitioning back to face-to-face learning in the last year. Acclimatization with all pandemic regulations that schools apply might increase the vulnerability to depression.

**Objectives:**

The current study aims to assess the levels of depression among Iraqi dentistry students after transitioning from online to onsite learning during the pandemic period.

**Methods:**

A cross-sectional study was conducted online after transitioning from online to the onsite learning method during the pandemic period. Sociodemographic data and Patient Health Questionnaire-8 (PHQ-8) were included in the questionnaire.

**Results:**

A total of 307 respondents, 216 (70.4%) female and 91 (29.6%) male, 276 (90%) live with family, 20 (6.5%) live with friends and 11 (3.5%) live alone, 268 (87.3%) of student claimed that post-COVID-19 regulations face to face learning is more stressful while 39 (12.7%) answered no difference. 39 (12.7%) of dentistry student with normal level of depression, 199 (38.8%) have mild depression 101 (32.9%) moderate depression, 32 (10.4%) moderately severe, 16 (5.2%) severe. Depression level and students’ perception of teaching mode transition showed a significant association (p<0.05).However, there are no significant associations between gender, living conditions, or dentistry stages with depression levels (p>0.05).

**Conclusions:**

A high prevalence of depression symptoms among Iraqi dentistry students was found during onsite learning, along with all educational institutions’ pandemic rules and regulations. Psychological supporting preventive programs are needed to apply for supporting students’ mental health.

**Disclosure of Interest:**

None Declared

